# Diagnostic Performance of SWE and Predictive Models Based on SWE for Post-Hepatectomy Liver Failure: A Systematic Review and Meta-analysis

**DOI:** 10.2174/0115734056379123250626163120

**Published:** 2025-07-02

**Authors:** Jiaxu Liang, Fukun Shi, Lan Zhang, Suo Yin, Yong Chen

**Affiliations:** 1 Department of Diagnostic Radiology, The Fifth Clinical Medical College of Henan University of Chinese Medicine (Zhengzhou People’s Hospital), Zhengzhou, China; 2 Department of MRI, The First Affiliated Hospital of Henan University of Chinese Medicine, Zhengzhou, China; 3 The First Clinical Medical College of Henan University of Chinese Medicine, Zhengzhou, China

**Keywords:** Shear wave elastography, Liver failure, Hepatectomy, Liver stiffness, Hepatocellular carcinoma, HSROC

## Abstract

**Background:**

Post-hepatic resection liver failure (PHLF) remains one of the most serious complications after hepatic resection, with an overall morbidity rate as high as 32% and an approximate 5% mortality. Previous studies demonstrate the potential of shear wave elastography (SWE) to predict PHLF. This meta-analysis aimed to evaluate the diagnostic accuracy of SWE in identifying liver failure after hepatectomy.

**Methods:**

A comprehensive search was performed across PubMed/Medline, Embase, and Web of Science to identify studies assessing the diagnostic accuracy of SWE for predicting PHLF. The combined sensitivity, specificity, and the hierarchical summary receiver operating characteristic curve (HSROC) for SWE in detecting PHLF in liver resection patients. The Quality Assessment of Diagnostic Accuracy Studies tool was used to evaluate the quality of the studies included in the analysis. Heterogeneity was explored through sensitivity analysis, univariable meta-regression and subgroup analysis.

**Results:**

This meta-analysis included a total of 13 studies involving 2985 patients. For quantitative analysis. The combined sensitivities and specificities of SWE for detecting post-hepatectomy liver failure were 0.81 and 0.68, respectively. The HSROC value for SWE was 0.82. Significant heterogeneity (I^2^ = 80.22) was observed in pooled specificity. Meta-regression and subgroup analyses suggest that differences in the proportion of patients with HCC and in the diagnostic criteria for PHLF may account for the observed heterogeneity. For the qualitative analysis, six predictive models based on SWE were included, and their AUCs were 0.80-0.915.

**Conclusion:**

Both SWE alone and SWE-based prediction models appear to accurately detect PHLF and help to categorize patients into high- and low-risk groups. It may also assist surgeons in identifying the best candidates for liver resection and enhancing perioperative management.

## 1 INTRODUCTION

Hepatocellular carcinoma (HCC) ranks among the most common malignant liver tumors globally [1]. Surgical resection, or hepatectomy, is the cornerstone of therapy for early-stage HCC [2, 3]. Post-hepatectomy liver failure (PHLF) is a serious post-hepatectomy complication caused by inadequate future liver remnant, impaired regenerative capacity, and altered hemodynamics, leading to metabolic dysfunction, coagulopathy, and potential multi-organ failure [4]. Despite significant advancements in surgical methods and tools that have markedly enhanced liver surgery, the occurrence of PHLF has been reported to reach up to 32%, accompanied by a mortality rate of 5% [5]. In clinical practice, evaluating the risk of PHLF prior to hepatectomy in patients with HCC is essential. Beyond conventional liver function tests such as bilirubin, albumin, and aspartate aminotransferase, various indices of liver function, such as the Child-Pugh score, albumin-bilirubin (ALBI) classification, Model for End-Stage Liver Disease (MELD) score, and indocyanine green clearance (ICG) assay, have been suggested to predict PHLF, however, ICG is not indicated for patients with iodine allergy or thyrotoxicosis, ALBI and MELD are dependent on indicators such as serum bilirubin and albumin, which can be affected by laboratory testing errors or changes in patient status, and have limited accuracy, with areas under the receiver operating characteristic curves (AUC) varying from 0.61 to 0.76 [6-9]. Therefore, more accurate preoperative evaluation and identification of patients at high risk for PHLF is critical, especially in patients with HCC or cirrhosis.

Recent findings have identified advanced fibrosis as a significant risk factor for PHLF following hepatic resection in patients with HCC. Previous research has indicated that liver fibrosis and cirrhosis negatively impact liver regeneration and contribute to the development of PHLF [10]. On the other hand, in up to 80% of cases, HCC is often associated with liver fibrosis or cirrhosis, leading to impaired liver function and an elevated risk of PHLF. Moreover, liver fibrosis biomarkers, such as serum markers and fibrosis models, have been used alone or together to evaluate liver fibrosis and predict PHLF. Many studies indicate that elastography is more accurate than other serum biomarkers in diagnosing hepatic fibrosis, therefore, liver stiffness based on elastography can be used as an adjunct to assess liver functional reserve and provide useful prognostic value for patients undergoing resection [11]. So far, Magnetic Resonance Elastography (MRE), Transient Elastography (TE), and Shear Wave Elastography (SWE) are the three most common elastography techniques [12, 13]. TE measures liver stiffness noninvasively and has therefore become a method for assessing liver fibrosis, but its applicability and accuracy are limited because of its own technical shortcomings, such as, it is unable to delineate the Region of Interest (ROI) [14]. Our previous meta-analysis of MRE for predicting complications following liver resection [15] highlighted its potential; However, this approach suffers from limited availability, time-consuming and low cost-effectiveness. SWE is a noninvasive technique recognized for its high reproducibility and effectiveness in accurately distinguishing significant fibrosis (F≥2) in patients with liver disease, making it the most commonly used method [16]. A recent meta-analysis showed that MRE and SWE also showed similar diagnostic accuracy in staging advanced fibrosis and cirrhosis in patients with chronic hepatitis B [17]. In other words, SWE may also possess a similar potential to MRE in predicting PHLF. Moreover, due to its own advantages, SWE is more likely to be widely used compared to MRE. Previous studies [18, 19] emphasized the potential value of liver stiffness measurements (LSM) using 2D-SWE in predicting PHLF; nevertheless, they are all based on small sample sizes, and the effectiveness of SWE in predicting PHLF has not been systematically assessed. To address this gap, we performed a meta-analysis to clarify the prognostic value of LSM measured by SWE alone and SWE-based predictive models for patients after hepatectomy.

## 2 METHOD

### 2.1 Materials and Methods

Our study was carried out following the guidelines of the Cochrane Handbook [20] and in accordance with the PRISMA-DTA Statement checklist (In supplementary materials) [21]. As this was a meta-analysis, ethic committee approval and informed consent were not required.

#### 2.1.1 Search Strategy

A comprehensive search for relevant citations on the use of SWE to detect LS for predicting PHLF was conducted. Two investigators (JXL and FKS) independently identified suitable studies published up to February 1, 2025. To gather all relevant published citations, multiple electronic databases (including PubMed/Medline, Embase, Web of Science databases.) were carefully searched. Full articles were available and written in English or Chinese. A combination of keywords and medical subject heading terms were used, including(((((“liver failure”) OR (“postoperative liver failure”)) OR (“Post-hepatectomy liver failure”)) OR (“PHLF”)) OR (“post-hepatectomy complication”)) AND (((((((“SWE”) OR (“shear wave elastography”)) OR (“2D-SWE”)) OR (“shear wave velocity”)) OR (“SWV”)) OR (“real-time elastography”)) OR (“RTE”)). Two researchers independently screened the search results and reviewed the full texts to determine inclusion. Grey literature or conference proceedings were excluded. EndNote (version X9) was used to manage the literature search results. Two researchers (JXL and FKS) independently screened titles/abstracts and assessed full text based on inclusion criteria. Discrepancies were resolved in a two-stage hierarchical agreement: First. Two-person primary check-off: Discrepancies and rationale were discussed until consensus was reached. Second: Unresolved conflicts were decided by Third researcher (LZ).

#### 2.1.2 Citation Selection

The initially identified articles underwent further evaluation. Two investigators independently selected references by examining the titles and abstracts of these papers. Full texts of studies that met the inclusion criteria were then acquired and scrutinized to determine their relevance. The final selection of target papers was collaboratively decided by two investigators.

All research included in this review needed to satisfy the following inclusion criteria: (1) study subjects were patients who underwent SWE measurement of background LS prior to major liver resection; (2) participants were aged 18 years or older; (3) the study evaluated the sensitivity, specificity and AUC of LS measured by SWE alone or prediction model based on SWE in detecting PHLF; and (4) the full text of the study was available.

Exclusion criteria were as follows: (1) previous liver transplantation; (2) absence of SWE measurements or technical failures in SWE; (3) LS assessment conducted by TE or MRE; (4) the study being a review, case report, or meta-analysis published in full text; and (5) animal studies. If the same cohort appeared in multiple journals, only the most comprehensive or informative studies were included to avoid duplication.

#### 2.1.3 Data Extraction and Quality Assessment

Two independent reviewers thoroughly examined the full texts of the studies. The gathered information included the first author’s name, study location, measuring instrument employed, publication year, patient age range, gender distribution (male/female), number of participants, study design, diagnostic criteria and proportion of HCC *etc*. The risk of bias and applicability concerns of the included cohorts were assessed by two independent readers using the QUADAS-2 tool. The quality assessment consisted of the evaluation of four components: patient selection, index tests, reference standard, and flow and timing bias risk was evaluated in each section. Discrepancies in data extraction were resolved through discussion. For quantitative analysis, we collected the TP, TN, FP, and FN results of SWE diagnosis from each study to calculate sensitivity and specificity. For qualitative analysis, we extracted only the components of each predictive model, its sensitivity, specificity and its AUC.

#### 2.1.4 Statistical Analysis

Using the extracted data, we calculated the overall sensitivity and specificity of SWE, along with their corresponding 95% confidence intervals (CIs), based on TP, TN, FP, and FN using STATA 16.0 software. Forest plots of sensitivity and specificity, as well as hierarchic summary receiver operating characteristic (HSROC) curves, were constructed to improve and extend the traditional summary receiver operating characteristic curve approach [22]. Heterogeneity was assessed using the Cochrane Q test and Higgins index of inconsistency (I^2^), where *p* < 0.05 or I^2^ > 50% indicated significant heterogeneity [23]. Diagnostic threshold effects were evaluated using the typical “shoulder-arm shape” of HSROC curve. Additionally, meta-regression and subgroup analysis were conducted to explore sources of heterogeneity. Covariates included the following: (1) diagnostic criteria (International Study Group of Liver Surgery (ISGLS)*vs*. non-ISGLS), (2) study population (Asian *vs*. no-Asian), (3) study design (prospective *vs*. retrospective), and (4) proportion of HCC (all *vs*. non-all), (5) SWE device (Supersonic *vs*. no-Supersonic). (6) HBV infection(>80% *vs*. <80%) sensitivity analysis was adopted to verify the robustness of the study results. A Fagan nomogram was utilized to evaluate the clinical utility of LSM by SWE in predicting PHLF in patients undergoing liver resection. Deeks funnel plot was employed to assess publication bias, with a P-value less than 0.10 indicating significant asymmetry.

## 3 RESULTS

### 3.1 Search Results and Study Characteristics

The flowchart depicting the study selection process is illustrated in Fig. (**1**). Twenty-two eligible full-text articles were assessed by removing duplicates and reading titles and abstracts. A total of 9 studies were excluded after reading the full text, among them, 1 study used a different index test, 2 were unable to extract data, 4 duplicated cohorts, and 2 studies about different study aims. Ultimately, 13 full-text studies were identified from the electronic searches, 9 studies can be quantitatively analyzed. It needs to be explained that in 1 of the studies, the abstract is in English while the full text is in Chinese [24]. Tables **1** and **2** detail the characteristics of the included studies and the characteristics of the patients in the different studies, respectively. A total of 13 studies [5, 18, 19, 24-33], involving 2985 patients (median age, 61.8 years, 83.9% male), were included in this meta-analysis. Among all included patients, 95.4% were HCC patients. 12 studies were conducted in Asia, and one in Europe. 8 studies had a prospective design, while the rest were retrospective. The findings from the study quality assessment are presented in Fig. (**2A** and **B**). Five included studies were of retrospective design., which may introduce unclear risks in patient selection. The differing diagnostic criteria for PHLF led to a high risk of bias in assessing the reference standard in the two studies included. Compared to other diagnostic criteria, ISGLS is widely regarded as the most standardized and clinically relevant method for diagnosing PHLF, which provides a set of internationally recognized standards that contribute to consistency across institutions and studies, improving the comparability of results.

### 3.2 Diagnostic Performance of SWE Alone and SWE-based Predictive Models for the Detection of PHLF

The bivariate random effects model can more reliably and accurately estimate the variability and heterogeneity between studies in meta-analyses by taking into account the differences between the results of different studies; therefore, this model was used in this study to assess the diagnostic accuracy test-related indicators of SWE for predicting PHLF. 9 Studies could be quantitative analysis of the diagnostic performance of SWE alone [18, 19, 24-30]. The combined sensitivity and specificity of SWE for detecting PHLF were 0.81 (95% CI, 0.75–0.86) and 0.68 (95% CI, 0.62–0.73) respectively, as shown in Figs. (**3** and **4a**). The combined Positive Likelihood Ratio (PLR), Negative Likelihood Ratio (NLR), and Diagnostic Odds Ratio (DOR) were 2.5 (95% CI: 2.2–3.0), 0.27 (95% CI: 0.21–0.36), and 9.0 (95% CI, 6.0–13.0) respectively. The heterogeneity of pooled sensitivity and specificity were I^2^ = 18.21% and I^2^ = 80.22%, respectively Fig. (**3**). Fig. (**4a**) presents the Hierarchical Summary Receiver Operating Characteristic curve (HSROC) was 0.82 (95% CI, 0.79–0.86). Notably, the HSROC plane did not exhibit a typical “shoulder-arm” shape, indicating the absence of a significant threshold effect. For the qualitative analysis, six predictive models were included [5, 19, 24, 31-33], and their sensitivity and specificity are presented in Table **3**, respectively. Moreover, their AUCs ranged from 0.80 to 0.915, which seems to be better than SWE alone, but some of these studies did not include a validation group.

### 3.3 Fagan Test

The Fagan nomogram is a graphical tool to visualize and understand diagnostic test results by combining the pre-test probabilities and likelihood ratios of the diagnostic results to calculate the post-test probabilities. In Fig. (**4b**), with the median prevalence of PHLF from the eligible studies, the pretest probability of 32% was assessed in relation to the post-test probabilities after obtaining a “positive” or “negative” result. With the summary sensitivity and specificity, the positive and negative post-probabilities of PHLF were determined to be 54% and 11% respectively.

### 3.4 Sensitivity Analysis

To account for the potential sources of heterogeneity, sensitivity analysis was conducted. The heterogeneity was still unchanged, and no significant difference was detected in the sensitivity analysis by omitting each of the included studies.

### 3.5 Univariate Meta-regression and Subgroup Analysis

Due to the significant heterogeneity in combined specificity among the included studies, we conducted meta-regression and subgroup analyses to determine the potential sources of them. Univariate meta-regression showed that the proportion of patients with HCC and the diagnostic criteria for PHLF were associated with pooled-specific heterogeneity (P<0.05, Fig. **4c**). The diagnostic performance of subgroup analysis was shown in Supplementary Table (supplementary material).However, the small sample size of the nine studies on LSM conducted through SWE limited the statistical power of the meta-regression, necessitating cautious interpretation of these results.

### 3.6 Publication Bias

Deek’s funnel plots to show the relationship between the study sample size and the effect estimate in the form of a scatterplot, which is used to check for the presence of reporting bias in the meta-analysis of diagnostic accuracy that its asymmetry may reveal publication bias or other differences in study quality. The funnel plot was relatively symmetrical, with *P* values of 0.15, showing no statistical significance (*P*>0.10), indicating the absence of significant publication bias (Fig. **4d**).

## 4 DISCUSSION

Although recent advancements in surgical techniques and post-operative care have improved clinical outcomes for liver resection, PHLF still significantly contributes to morbidity and mortality after the procedure [34, 35]. Liver fibrosis is usually caused by hepatocyte injury and has a major influence on the prognosis of individuals with chronic liver disease. Therefore, a more accurate assessment of hepatic fibrosis could enhance the prediction of liver reserve function, which in turn enhances the prediction of PHLF occurrence. Our focus was on the effectiveness of preoperative liver fibrosis evaluation in predicting PHLF. Recently, advanced elastography methods such as TE, SWE, and MRE have proven valuable in identifying PHLF [12]. SWE is the most popular method in general medical settings for accurately measuring tissue stiffness. This technology is instantaneous, non-invasive, and easy to use [14].

To the best of our knowledge, this study represents the first meta-analysis of the application of SWE to detect PHLF. In this meta-analysis involving 13 studies and 2985 patients, the overall sensitivity, specificity, and AUC of using SWE alone for preoperative LSM to predict PHLF were 0.81 (95% CI, 0.75-0.86), 0.68 (95% CI, 0.62-0.73), and 0.82 (95% CI, 0.79-0.86), respectively. Our study findings suggest that SWE has a high sensitivity, LSM by SWE serves as a significant predictor of postoperative outcomes following hepatectomy, which indicates that 2D-SWE images can effectively stratify patients into high-risk and low-risk groups for symptomatic PHLF, Assessing the risk of PHLF prior to hepatic resection in patients with HCC, especially those with cirrhosis, is critical to identify suitable candidates for hepatic resection and to exclude those who can be managed with other therapies such as transplantation, transarterial chemoembolization, and thermal ablation. This helps surgeons identify the best candidates for liver resection and enhance perioperative management, but we have not established a widely accepted cut-off value. However, its specificity is not high, probably because PHLF is caused by a variety of factors, not solely determined by the only factor, liver stiffness, including other factors such as abnormal liver function and clinically significant portal hypertension (CSPH). Combining these clinical indicators with SWE can effectively improve the low specificity of SWE and thus reduce the incidence of false positives. Several studies have shown that liver stiffness, in combination with liver function tests and clinical information, is a better predictor of the risk of serious complications in patients undergoing hepatectomy for HCC than LSM alone [19, 24]. Nomogram is an easy-to-calculate visualization chart, and in recent years, several Nomogram containing SWE have been introduced [5, 31, 32]. Among them, Long *et al*. [5] recently created a predictive model that includes factors such as FLR ratio (future liver remnant liver volume), LSM with SWE≥ 9.5 kPa, Child-Pugh classification, and presence of CSPH. This nomogram had the best diagnostic performance so far (AUC=0.915). But all of the above-mentioned prediction models were constructed through traditional logistic regression analysis. With the continuous development of radiomics, machine learning, and Deep learning, new algorithms are emerging constantly for constructing prediction models [15, 36, 37]. These methods related to artificial intelligence are used for the diagnosis of various diseases, including tumors, cardiovascular diseases, skin diseases, *etc*, and all have demonstrated excellent diagnostic performance. Univariate meta-regression showed that the diagnostic criteria for PHLF and the proportion of patients with HCC were the source of heterogeneity (I^2^ = 80.22%) in pooled specificity. Different from the Clavien-Dindo classification and the Comprehensive Complication Index (CCI) [38, 39], which are evaluation systems for general postoperative complications the criteria established by the ISGLS are specific standards formulated for hepatic surgery, which may account for this discrepancy. On the other hand, the pooled specificity was relatively lower in the studies that only included patients with HCC. This may be explained by the fact that PHLF in HCC patients was not only determined by liver fibrosis but also associated with other clinical factors. Moreover, it also accounts for why the predictive model constructed with multiple factors was superior to SWE alone.

Studies have also shown that SWE better identifies and grades PHLF compared to traditional assessment methods. Fu *et al*. [29] demonstrated that SWE had a higher AUC for the prediction of PHLF (AUC = 0.795) than the ICG-R15 (AUC = 0.619) and the ALBI score (AUC = 0.686), and a higher specificity for the prediction of PHLF (76.3%) than the ICG-R15 (66.1%) and the ALBI score (69.5%); In another study [29] diagnosing symptomatic PHLF, SWE showed similarly better diagnostic accuracy compared to ICG-15, especially in PHLF grades B and C. Moreover, liver stiffness is a key feature that directly reflects the liver's condition, unlike other indirect indicators. Regarding elastography modalities, TE was the first widely used ultrasound elastography technique. A comparative study [30] demonstrated that the diagnostic performance of 2D-SWE was significantly better than that of TE in predicting serious complications after hepatectomy for liver tumors (AUC: 0.854 for 2D-SWE and 0.692 for TE, P = 0.004). Compared with 2D-SWE, one drawback of TE is its inability to provide real-time B-mode grayscale liver images, which complicates the precise localization of the measurement area within the liver tissue and fails to avoid measurement inaccuracies caused by large hepatic vessels or liver lesions. In addition, 2D-SWE can be converted to a standard B-mode examination. It also alleviates the challenges posed by large tumors in the right liver lobe that may prevent LS measurements using TE [40]. Moreover, TE is also limited in patients with ascites, severe obesity, or thick subcutaneous adipose tissue in the abdomen [5]. Consequently, 2D-SWE provides more reliable and accurate liver stiffness measurements than TE. Our group previously conducted a meta-analysis on MRE to predict postoperative complications, with an AUROC of 0.83 (95% CI: 0.80-0.86) [15]. Although MRE has slightly higher diagnostic performance than SWE, it is more challenging to implement due to its higher cost, longer time-consuming and lower availability. Consequently, it is essential to conduct large-scale studies that directly compare the diagnostic performance of SWE with these two elastography modalities.

This study encountered several limitations. Firstly, SWE is a novel technology that has not been widely studied for PHLF detection, resulting in a relatively small patient sample in the included studies, which limits its use in clinical. Second, five of the included studies were retrospective in design, and the combined sensitivity and specificity of retrospective studies were higher than those of prospective studies in the subgroup analyses, possibly related to the nature of retrospective studies, which are likely to be biased in patient selection. Thirdly, most of the research were carried out in Asia, which may have contributed to selection bias due to the high incidence of hepatitis B virus infection in the region, resulting in a significant proportion of participants having HBV-related HCC, which may limit the applicability of SWE to with varying liver disease etiologies, such as hepatitis C virus (HCV) infection or non-alcoholic fatty liver disease (NAFLD). Fourth, our results include 9.3% non-HCC patients, a very small proportion, but which introduces some uncertainty into our results. However, since this was a study-level Meta-analysis, we were unable to obtain individual patient data, thus completely eliminating this confounding. Finally, two of the studies used different diagnostic criteria for PHLF, which could have had a potential impact on the determination of the final outcome, and we identified them as high-risk in the QUADAS-2 tool. On the other hand, these different diagnostic criteria and proportions of HCC patients were found in meta-regression analyses as possible sources of heterogeneity. Future studies should include more cohorts with standardized protocols, such as patients with the same etiology in different races and using the same reference standard, ISGLS, to enhance comparability between studies and improve consistency of results. In summary, more prospective studies in broader and more homogeneous populations are needed to clarify the role of SWE in predicting PHLF.

## CONCLUSION

In conclusion, LSM by SWE has significant predictive value for symptomatic PHLF after HCC resection in the Asian population. It has unique advantages over other elastography techniques, including low cost, time efficiency, and easier availability, which further facilitate its widespread clinical application. In addition, a new predictive model combining LS by SWE with clinical and serum biomarkers may improve the ability to pinpoint patients at high risk for complications after hepatectomy, helping clinicians make safe and reasonable clinical decisions regarding surgical indications and perioperative management in their daily practice.

## Figures and Tables

**Fig. (1):**
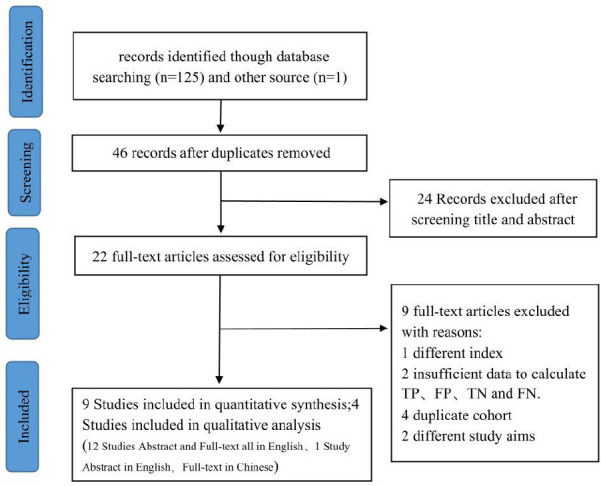
PRISMA flow diagram of primary studies included in both the present systematic review and meta-analysis. **Abbreviation:** TP: true positive; TN: true negative; FP: false positive; FN: false negative.

**Fig. (2):**
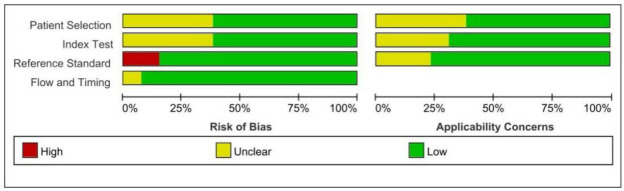
Quality assessment with QUADAS-2 criteria. (**A**). Risk of bias graph and applicability concerns: review authors’ judgements about each domain presented as percentages across included studies.

**(B):**
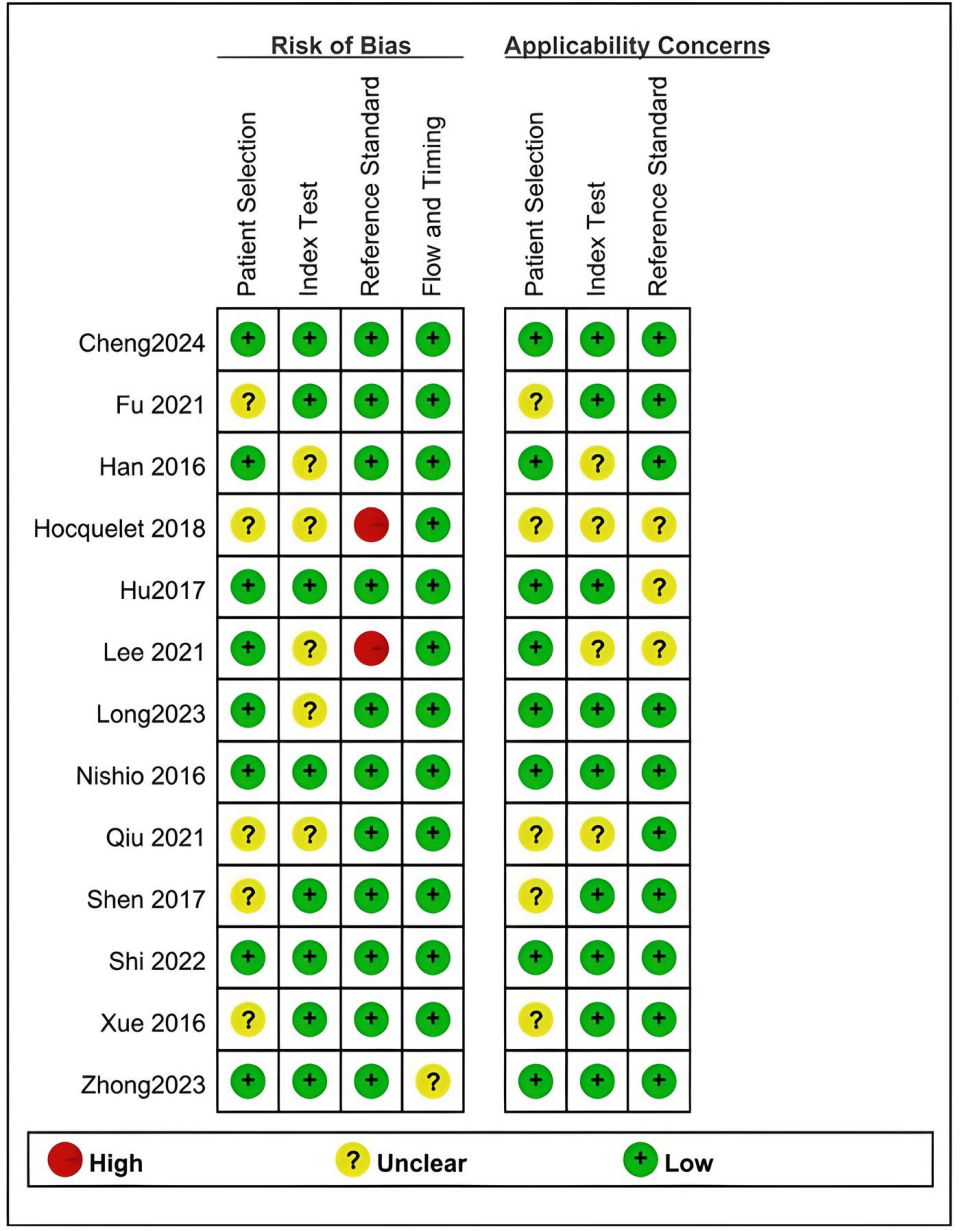
Summary of bias risk and applicability concerns: review authors’ judgments about each domain for each included study.

**Fig. (3):**
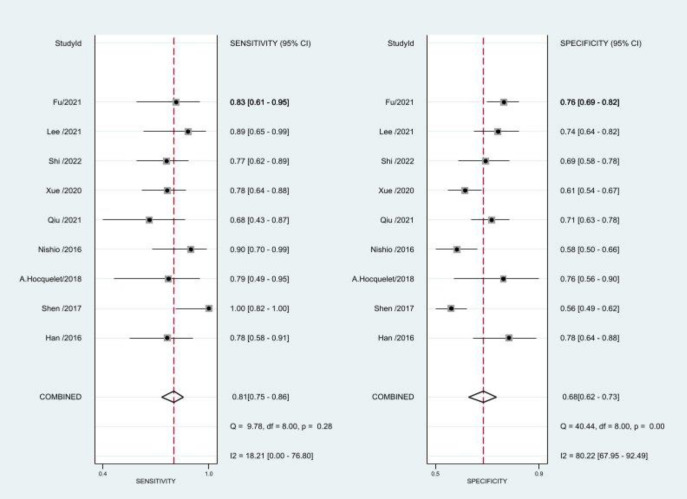
Pooled sensitivity and specificity were employed to assess the predictive capability of shear wave elastography (SWE) in identifying post-hepatectomy liver failure (PHLF).

**Fig. (4a):**
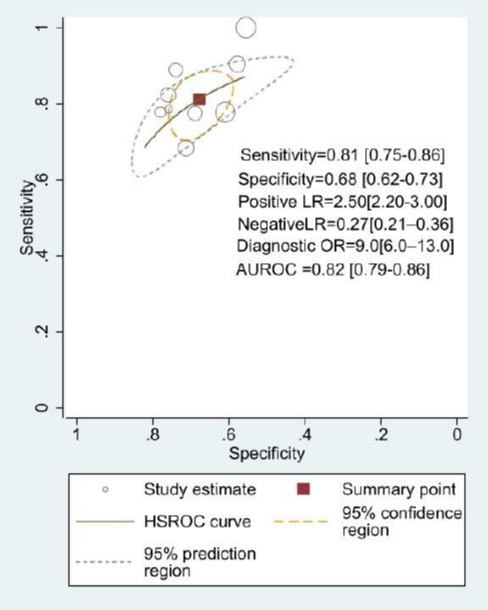
Hierarchic summary receiver operating characteristic (HSROC) curves and test performance to detect the diagnostic performance of shear wave elastography (SWE) for predicting post-hepatectomy liver failure (PHLF). A bivariate random effects model was used to estimate the sensitivity and specificity for liver stiffness measured using SWE. **Abbreviation:** Positive LR: Positive Likelihood Ratio; Negative LR: Negative Likelihood Ratio; Diagnostic OR: Diagnostic odds ratios.

**Fig. (4b):**
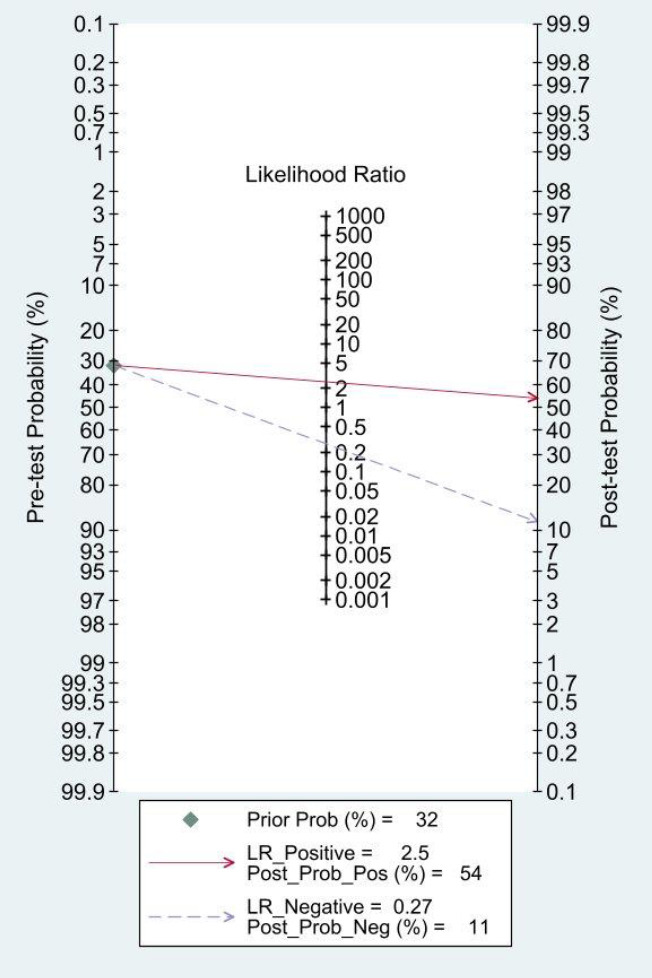
Fagan’s nomogram was used to assess the post-test probabilities. When the test result was positive, the probability of accurate detection increased from 32% (pre-test probability) to 54% (post-test probability). When the test result was negative, the probability of accurate detection decreased from 32% (pre-test probability) to 11% (post-test probability), further indicating that SWE can improve the diagnostic efficiency of PHLF. **Abbreviation:** Prior Prob: Prior Probability; LR-Positive: Positive Likelihood Ratio; Post-Pro-Pos: Post- Probability- Positive; LR-Negative: Negative Likelihood Ratio; Post-Pro-Neg: Post-Pro-Neg: Post-Probability- Negative.

**Fig. (4c):**
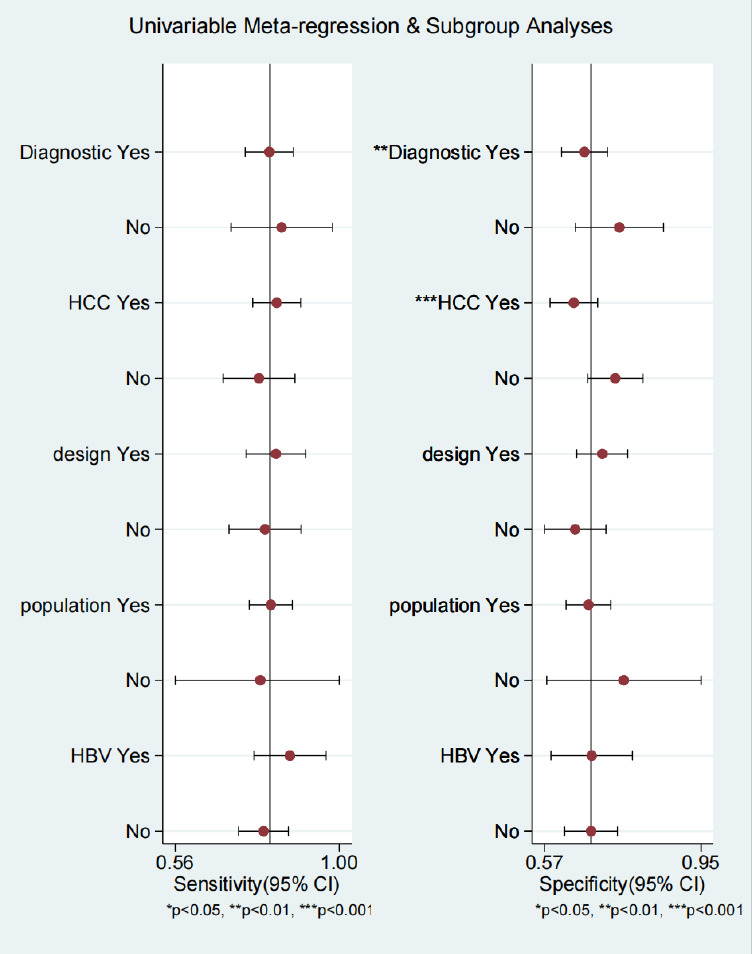
Univariate meta-regression and subgroup analysis of the diagnostic performance of SWE for detecting PHLF. Diagnostic yes = use the International Study Group of Liver Surgery (ISGLS) criteria for diagnosing PHLF as the control group; HCC -yes = All patients included were Hepatocellular carcinoma (HCC) as the control group; population yes = Patients included were from Asia as the control group; design yes = Prospective study as control group; SWE yes = SWE device uses the Supersonic. HBV yes = >80% of patients are HBV-infected as the control group.

**Fig. (4d):**
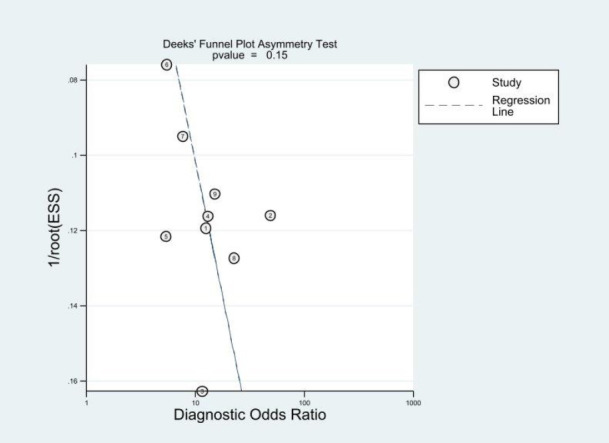
Deeks’ Funnel plots of diagnostic odds ratios for SWE study results did not demonstrate a risk of publication bias (*P* = 0.15).

**Table 1:** Characteristics of the included studies.

**Authors**	**Year**	**Country**	**Duration**	**Study-design**	**SWE Model**	**Reference Standard**
Han	2016	China	Jan 2014-Dec 2015	prospective	Philips EPIQ7	ISGLS
Shen	2017	China	July 2015-July 2016	retrospective	Supersonic	ISGLS
Hocquelet	2018	France	2013-2015	retrospective	Siemens	Dindo—Clavien classification
Nishio	2016	Japan	Aug 2011-Oct 2014	prospective	Siemens	ISGLS
Xue	2020	China	Jan 2015-Jan 2016	retrospective	Supersonic	ISGLS
Lee	2021	Korea	Jun 2018-Nov 2019	prospective	Aplio i900	CCI
Qiu	2021	China	Dec 2016-Feb 2018	retrospective	Supersonic	ISGLS
Shi	2022	China	Aug 2018-July 2021	prospective	Supersonic	ISGLS
Fu	2021	China	Feb 2019-Dec 2019	retrospective	Supersonic	ISGLS
Cheng	2024	China	Oct 2020-Mar 2022	prospective	Supersonic	ISGLS
Hu	2017	China	Oct 2020-Mar 2022	prospective	Philips	ISGLS
Long	2023	China	Aug 2018-Apr 2021	prospective	Supersonic	ISGLS
Zhong	2023	China	Aug 2018-Oct 2022	prospective	Supersonic	ISGLS

**Abbreviation:** ISGLS: International Study Group of Liver Surgery.

**Table 2:** Patient’s characteristics of included studies.

**Author**	**Year**	**Age**	**Male/Female**	**HCC**(**%**)	**ALT(U/L)**	**AST(U/L)**	**Albumin(g/L)**	**Platelet count(10^9^/L)**	**F3-4(%)**	**Child-Pugh grade(%)**	**MELD score**	**HBV infection**
Han	2016	59(24-91)	63/14	52(65%)	30(8-98)	31(14-167)	41(26-53)	173(50-606)	F4:48(60)	A:72(90)	5.8(4.8-6.5)	69 (89.6%)
Hocquelet	2018	66(39-85)	43*	23(53.5%)				218 (107—595)	F3:5(11.6) F4:8(18.6)			
Nishio	2016	68 ±10	140/37	177(100%)		35(16-234)	38 ± 4	152±58	F3:30(17.6) F4:41(24.1)			36(20.3%)
Lee	2021	61(53-69)	83/42	95(76.0%)			41(39–44)	180.0(139.0–210.5)	F3:29(23.2) F4:40(32.0)			74 (59.2%)
Qiu	2021	54±12	135/37	111(64.5%)	28(20-41)	32(32-41)		131 (84–184)				
Shi	2022	53.9± 11	118/12	130(100%)	33(13-162)	35(13-168)	38.7(27.9–48.7)		F3:31(23.8) F4:31(23.8)	A:121(93.1) B:9(6.9)	4.37(-3.19-13.00)	127(97.7%)
Fu	2021	2021	179/36	215(100%)		34	42.2	137	F3:52(24.2) F4:100(46.5)	A:215(100)		
Shen	2017	56.10±10.89	240/40	280(100%)	29.43 (146)/ 34(149)^∆^			165.25(560)/ 143(234)^∆^	F3:34(12.1) F4:141(50.4)	A:274(97.9) B:6(2.1)	7.08 ± 1.35 7.65 ± 1.03^∆^	227(81.1%)
Xue	2020	56 ± 11	235/39	274(100%)	30(6-152)/ 33.5(10-131)^∆^		42.5(22-67)/ 40(33-50)^∆^	164(46-606)/ 143.5(36.0-270.0)^∆^	F3:34(19.5) F4:138(50.4)	A:269(98.2) B:5(1.8)	6.6(6.4-20.5)/ 7.5(6.4-10.4)^∆^	
Cheng	2024	54.4±10.7	458/104	562(100%)	27(19.0-38.0)/ 32(21.0-39.0) ^∆^		43(40.0-46.0)/ 41(38.8-45.0) ^∆^	148.5(111.0-197.0)/ 167.5(139.8-192.0) ^∆^				528(94.0%)
Hu	2017	56.5±10.5	177/39	216(100%)	57.8±80.6/ 50.7±47.9 ^∆^	60.6±84.4/ 54.9±53.4^∆^	38.9±6.7/ 40.3±7.1^∆^	166±83/158±71^∆^	F4:113(52.3)	A:192(88.9) B:24(11.1)	7.0±1.3/6.7±1.3^∆^	192(88.9%)
Long	2023	55.5(45-64)	201/22	223(100%)	32(21-40)/ 35(19-55) ^∆^		38.4(36.2-41.0)/ 38.2(35.9-41.4) ^∆^	199(154-241)/ 203(143-271) ^∆^		A:211(94.6) B:12(5.4)	4.57(2.48-6.14)/ 4.84(2.60-6.34) ^∆^	215(96.4%)
Zhong	2023	55.0(47.0-64.0)	305/40	345(100%)	31.0(21.0-43.5)	31.0(21.0-43.5)	38.3(36.2-41.0)			A:329 B:16	4.8(2.9-6.3)	330(95.7%)

Data are expressed in mean ± standard deviation, or in median (range), or n (%). BMI, body mass index; HCC, hepatocellular carcinoma**;** ALT, alanine aminotransferase; AST, aspartate transaminase; F3-4, Fibrosis stage 3-4**;** MELD, model of end-stage liver disease; *As male/female ratio could not be calculated, it is shown as a total number of people; ^∆^ means no PHLF/PHLF or Training cohort/ Validation cohort.

**Table 3:** Predictive effect of study's model based on SWE and clinical data on PHLF.

**Study**	**Variable**	**AUC(95%CI)**		**Sensitivity(%)**		**Specificity(%)**	
		**Training Cohort**	**Validation Cohort**	**Training Cohort**	**Validation Cohort**	**Training Cohort**	**Validation Cohort**
Cheng	INR+TB+BL+RR+SPA+LS	0.833(0.792,0.873)	0.802(0.684-0.920)	83.1	95.5	73.5	52.5
Hu	PT+SB+sGGT+CSPH+ElastPQ	0.85(0.784,0.917)	0.824(0.729,0.918)				
Long	FLR /TLV+CP+LS(>9.5)+CSPH	0.915	0.876(0.754,1.000)	78.0		91.5	
Zhong	CSPH+INR+ FLR /TLV+Radiomics**_-_**score	0.867(0.787,0.947)	0.822(0.720,0.898)	80	70.4	80	77.3
Xue	LS+INR+LN	0.82(0.76,0.89)	0.81	87.0		70.0	
Nishio	Rem+LS	0.80(0.70,0.87)		95.0		60.0	

**Abbreviations:** INR: International normalized ratio, multiplied by 10; TB: Total bilirubin; BL: Blood loss; RR: Range of resection; SPA: Spleen area; LS: Liver stiffness; PT: Platelet count; SB:Serum bilirubin; sGGT: serum gamma-glutamyl transferase; CSPH: Clinical signs of portal hypertension; ElastPQ: Elast point quantitative; FLR /TLV: future liver remnant/total liver volume; CP: Child Pugh; Emin: minimum of tissue elasticity; LN: Laminin; Rem: remnant liver volume rate.

## Data Availability

All data generated or analyzed during this study are included in this published article (and its supplementary information files).

## LIST OF ABBREVIATIONS

MELD: Model for End-Stage Liver Disease
ICG: Indocyanine Green Clearance
HSROC: Hierarchical Summary Receiver Operating Characteristic Curve
SWE: Shear Wave Elastography
PHLF: Post-hepatic Resection Liver Failure
TE: Transient Elastography
MRE: Magnetic Resonance Elastography
ROI: Region of Interest
CSPH: Clinically Significant Portal Hypertension

## STANDARDS OF REPORTING

PRISMA guidelines and methodology were followed.
